# Glucose control of glucagon secretion—‘There’s a brand-new gimmick every year’

**DOI:** 10.3109/03009734.2016.1154905

**Published:** 2016-03-24

**Authors:** Erik Gylfe

**Affiliations:** Department of Medical Cell Biology, Uppsala University, Uppsala, Sweden

**Keywords:** Diabetes, glucagon secretion, glucose homeostasis, signal transduction

## Abstract

Glucagon from the pancreatic α-cells is a major blood glucose-regulating hormone whose most important role is to prevent hypoglycaemia that can be life-threatening due to the brain’s strong dependence on glucose as energy source. Lack of blood glucose-lowering insulin after malfunction or autoimmune destruction of the pancreatic β-cells is the recognized cause of diabetes, but recent evidence indicates that diabetic hyperglycaemia would not develop unless lack of insulin was accompanied by hypersecretion of glucagon. Glucagon release has therefore become an increasingly important target in diabetes management. Despite decades of research, an understanding of how glucagon secretion is regulated remains elusive, and fundamentally different mechanisms continue to be proposed. The autonomous nervous system is an important determinant of glucagon release, but it is clear that secretion is also directly regulated within the pancreatic islets. The present review focuses on pancreatic islet mechanisms involved in glucose regulation of glucagon release. It will be argued that α-cell-intrinsic processes are most important for regulation of glucagon release during recovery from hypoglycaemia and that paracrine inhibition by somatostatin from the δ-cells shapes pulsatile glucagon release in hyperglycaemia. The electrically coupled β-cells ultimately determine islet hormone pulsatility by releasing synchronizing factors that affect the α- and δ-cells.

## Introduction

After Paul Langerhans’s discovery of the pancreatic islets in 1869 (1), Diamare found in 1899 that they contain two types of cells (2), which Lane in 1907 designated as α- and β-cells (3) (later also A and B cells). In 1915 Homans predicted that the B cells are the origin of ‘an internal secretion vital to the utilization of dextrose’ (4), and the glucose-lowering hormone, insulin, was discovered by Banting and Best in 1921 (5). In 1923 Murlin et al. observed that pancreatic insulin extracts sometimes were contaminated with a hyperglycaemic factor (6) that was named glucagon (7), whose production was eventually linked to the α-cells by Sutherland and De Duve in 1948 (8). Claes Hellerström, who is honoured in the current issue of this journal, made significant contributions in the glucagon field in his early scientific career. Together with his mentor Bo Hellman, Hellerström invented a silver impregnation technique that revealed A cell heterogeneity (9). The silver-positive and -negative A cells were named A_1_ and A_2_, respectively. They also provided indirect evidence that glucagon originates from the A_2_ cells by showing that they undergo pronounced nuclear atrophy after glucagon injections (10). Hellerström later pioneered studies of glucagon biosynthesis in guinea pig islets (11) as well as studies of metabolism (oxygen consumption) in A cell-enriched islets from streptozotocin-diabetic guinea pigs (12). Hellman and Lernmark showed that the α_1_ or A_1_ cells, which later proved to be identical to the D cells defined by Bloom in 1931 (13), secrete a factor that inhibits insulin secretion (14). This D cell (δ-cell) factor was later identified as somatostatin (15). There is still some confusion with regard to the use of the Greek or Latin designations, but the glucagon-producing cells are nowadays mostly referred to as α-cells and those secreting insulin and somatostatin as β- and δ-cells, respectively, which is the nomenclature subsequently used in this review.

With the discovery of insulin, life-saving treatment of diabetic patients rapidly became possible (16). It is consequently not surprising that most research has been focused on insulin and its actions in the search for improved strategies for optimizing blood glucose control and preventing secondary diabetes complications. Although it has become increasingly clear that hypersecretion of glucagon contributes to hyperglycaemia in diabetes (see (17) for review), it may have surprised many when the Unger group provided evidence that lack of insulin does not lead to diabetes in mice if the blood glucose-elevating effect of glucagon is prevented (18,19). However, disruption of glucagon action/secretion did not improve glucose tolerance in diabetic mice in another study (20).

Whereas diabetic hyperglycaemia may persist undetected for long periods of time, severe hypoglycaemia is acutely life-threatening due to the brain’s strong dependence on glucose as energy source. Glucagon is the major glucose counter-regulatory hormone, and its physiologically most important role is to prevent hypoglycaemia. Glucagon secretion is consequently stimulated when the blood sugar concentration falls below the resting level. Unfortunately, also this mechanism is compromised in diabetes (21), implying deteriorated recovery from dangerous hypoglycaemia that may occur accidentally in patients subjected to aggressive glucose-lowering treatment to reduce diabetes complications. Being essential for survival and well-being, the release of insulin and glucagon is controlled by multiple direct and indirect mechanisms. The blood glucose concentration is e.g. monitored by glucose-sensing cells in the portal vein area and in different regions of the brain, resulting in parasympathetic stimulation of insulin release in hyperglycaemia and glucagon release in hypoglycaemia, as well as sympathetic inhibition of insulin and stimulation of glucagon secretion in hypoglycaemia (22,23). However, the contribution of neural influence may differ between species since human islets are less innervated than rodent islets (24). Moreover, it is clear that glucose control of insulin and glucagon secretion persists in the perfused pancreas and isolated pancreatic islets. The subsequent focus will be on the glucose regulation of glucagon release that occurs within the pancreatic islets.

Already 30 years ago there was a unifying theory how the β-cells recognize glucose and the subsequent signalling that triggers release of insulin (25). The major aspects of the so-called consensus hypothesis are still valid and imply that glucose is rapidly taken up by the β-cells and metabolized to generate ATP. The resulting increase of the ATP/ADP ratio closes ATP-sensitive K^+^ (K_ATP_) channels to depolarize the β-cell and open voltage-dependent L-type Ca^2+^ channels. Subsequent influx of Ca^2+^ raises the cytoplasmic Ca^2+^ concentration ([Ca^2+^]_i_), which is the most important trigger of insulin release. This model consequently implies that the β-cells have an intrinsic capacity to sense glucose and release insulin as required, but [Ca^2+^]_i_-triggered secretion can also be enhanced or reduced by increase or decrease of cAMP, as well as by modulation of protein kinase C and the protein phosphatase calcineurin, mediated by paracrine effects of α-cell glucagon, δ-cell somatostatin and neurotransmitters like acetylcholine and noradrenaline (26,27).

**Figure 1.**
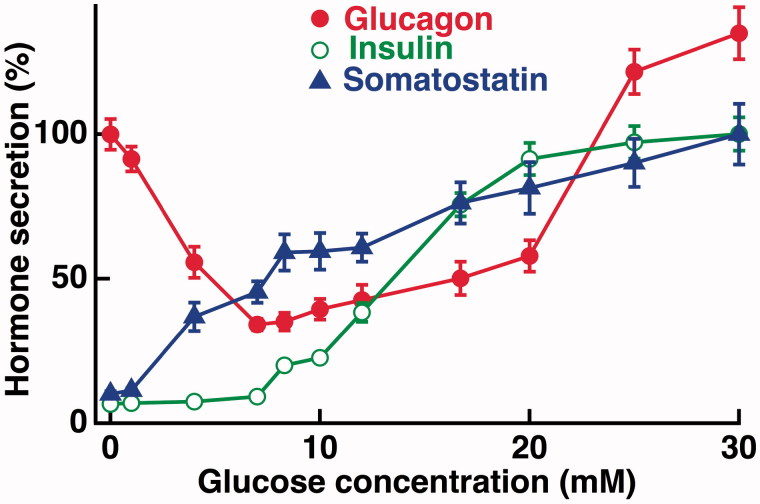
Dose–response relationships for glucose-regulated secretion of glucagon, insulin, and somatostatin. Batches of mouse islets were incubated for 60 min with glucose concentrations ranging from 0 to 30 mM. Glucagon secretion is expressed in per cent of that stimulated by absence of glucose, whereas insulin and somatostatin secretion are expressed in per cent of that stimulated by 30 mM glucose. Mean values ± SEM for eight experiments. The illustration is essentially based on data, from Vieira et al. 2007 (40), combined with data at 25 and 30 mM glucose that were not included in the original publication. Glucagon secretion at >20 mM glucose exceeds that stimulated by the absence of sugar.

The understanding of how glucose regulates glucagon secretion from the α-cells has progressed much more slowly, and it is remarkable that fundamentally different mechanisms continue to be proposed. Although Ca^2+^ and cAMP are generally believed to have similar secretion-triggering and -amplifying functions for glucagon as for insulin secretion (28–33), recent data indicate that glucagon release may sometimes be controlled independently of [Ca^2+^]_i_ (34–36). Lack of paracrine inhibition of the α-cells is often considered to underlie glucagon hypersecretion in diabetic hyperglycaemia, and paracrine factors have also been implicated in glucose control of glucagon secretion in hypoglycaemia. However, the spectrum of putative paracrine factors released from adjacent islet cells clearly differs under hypo- and hyperglycaemia due to specific glucose sensitivities of the different islet cell types (Figure 1). This review will therefore consider how glucose controls glucagon secretion by α-cell interactions with other islet cells as well as by α-cell-intrinsic mechanisms that contribute differently depending on the prevailing glucose concentration (Figure 2). The present contribution extends and updates a previous review with similar focus on glucose (37) but is more limited than another review (38) with regard to other modulators of glucagon release.

**Figure:**
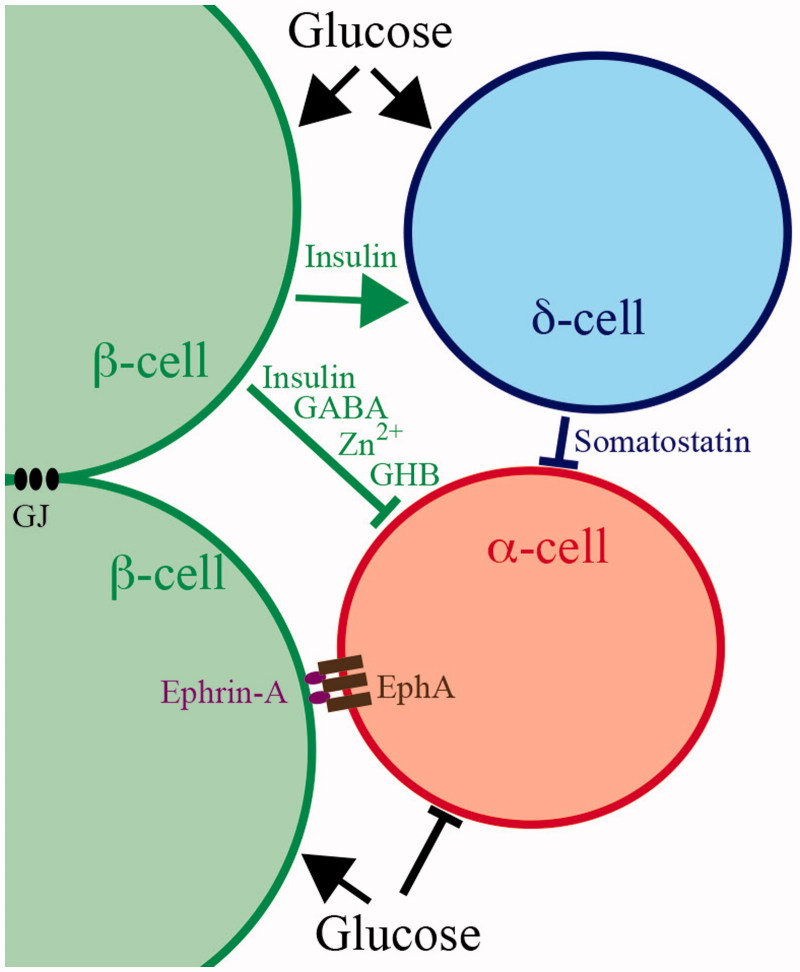
Figure 2. Models for local glucose regulation of glucagon secretion within pancreatic islets. Only the β-cells are electrically coupled by gap junctions (GJ). When stimulated by glucose they release insulin, γ-aminobutyric acid (GABA), Zn^2+^, or γ-hydroxybutyric acid (GHB), which have been implicated to mediate inhibition of glucagon secretion from the α-cells. β-Cell-mediated inhibition of the α-cells could also involve juxtacrine ephrin-A–EphA forward signalling. The δ-cells are stimulated by glucose and probably by insulin to release somatostatin that inhibits glucagon release from the α-cells. In addition, glucose has direct inhibitory effects on glucagon release by? -cell-intrinsic mechanisms.

## Autocrine amplification of glucagon release

Mouse and human pancreatic islets exposed to hypoglycaemic conditions increase their release of glucagon with maximal effect in the complete absence of sugar (39–41), although there should be less energy available to fuel α-cell secretion under such conditions. Positive feedback has been proposed to potentiate secretion in this situation and to explain how relatively modest changes in blood glucose can guarantee appropriate regulation of glucagon release (42). Indeed, glucagon has been found to amplify its own secretion by raising α-cell cAMP (32,33), and glutamate co-released with glucagon can promote secretion by raising [Ca^2+^]_i_ after autocrine activation of AMPA/kainate receptors (42).

## Paracrine control of glucagon release

### Inhibition by β-cell factors

Insulin released from the β-cells was first proposed (43) and is still considered as a putative mediator of glucose-inhibited glucagon secretion (18,44). Insulin may activate ATP-sensitive K^+^ (K_ATP_) channels to hyperpolarize the α-cells and close voltage-dependent Ca^2+^ channels (45), increase the expression of inhibitory γ-aminobutyric acid (GABA) A receptors (see below) (46), or induce cAMP degradation after activation of phosphodiesterase 3B (44). Other studies proposed that Zn^2+^ co-released with insulin mediates the inhibition (47,48), which involves K_ATP_ channel activation and hyperpolarization of the α-cells (45,49). Release of ATP from the secretory granules has also been implicated in paracrine inhibition of α-cell Ca^2+^ signalling and glucagon secretion from mouse islets (50). Despite an anticipated Ca^2+^-elevating effect of ATP, this inhibition of secretion was somehow proposed to involve activation of P2Y_1_ purinoceptors. This finding is contradicted by the observation in rat islets that blockade of this receptor inhibits rather than stimulates glucagon release (51). Other putative inhibitory β-cell-derived messengers are GABA (52) and its metabolite γ-hydroxybutyric acid (GHB) (53). GABA, which inhibits glucagon release by activating a hyperpolarizing Cl^-^ influx in α-cells (52), is present in β-cell synaptic-like microvesicles and insulin-containing granules that undergo exocytosis in response to glucose stimulation (54,55). However, there is also non-vesicular GABA (55,56), and total release was actually reported to be inhibited by glucose (56). GHB, whose release by an unknown mechanism is stimulated by glucose, acts on putative inhibitory receptors on the α-cells (53).

Most evidence that paracrine β-cell factors mediate glucose inhibition of glucagon release comes from experiments comparing hypoglycaemic (<3 mM) and hyperglycaemic (>8 mM) concentrations of glucose. However, maximal inhibition of glucagon release from mouse and human islets is reached already at 5–7 mM glucose (39–41), which corresponds to normal glycaemia in these species (Figure 1). A general problem with the idea that glucose-induced release of insulin and factors co-released with insulin mediate inhibition of glucagon secretion is that insulin release from mouse islets is only stimulated by glucose concentrations higher than 5–7 mM (Figure 1) (39–41). Although the human threshold for insulin secretion is somewhat lower (41), glucagon release is regulated by glucose also below this threshold. Exogenously administered insulin has nevertheless been found to inhibit glucagon secretion from human islets exposed to 1 mM glucose (41), but an insulin receptor antagonist had no effect on glucagon secretion from mouse islets exposed to 3 mM glucose (36). If basal insulin release affects glucagon secretion under hypoglycaemic conditions its role may be permissive rather than regulatory. It cannot be excluded that release of a putative inhibitory β-cell factor is controlled by lower glucose concentrations than those regulating insulin secretion. However, evidence is currently lacking that β-cell exocytosis of different types of vesicles shows separate glucose dependencies (54). The possibility remains that non-vesicular release of inhibitory factors may be controlled by glucose concentrations lower than those triggering exocytosis. In the case of GABA, such control does not seem to mediate inhibition of glucagon secretion. As mentioned above, total GABA release is inhibited rather than stimulated by glucose (56). While 5 mM glucose was found to stimulate GHB release from human islets (53), it remains to be established if secretion is differently regulated than that of insulin, since this glucose concentration is above the threshold for insulin secretion from human islets.

**Figure 3.**
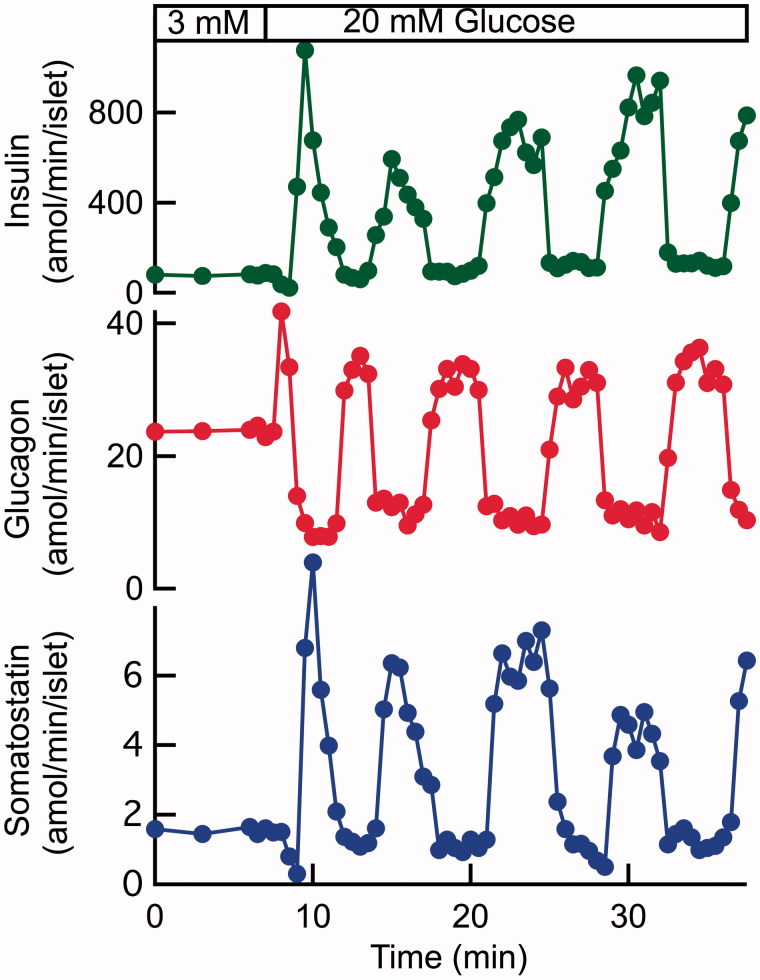
An increase of the glucose concentration from 3 to 20 mM induces pulsatile release of insulin, glucagon, and somatostatin from a batch of perifused human pancreatic islets. The insulin and somatostatin pulses are synchronized in the same phase and the glucagon pulses in opposite phase. The increase of the glucose concentration stimulates time-average release of insulin (360%) and somatostatin (105%) and inhibits that of glucagon (16%). The peaks of glucagon secretion exceed secretion at 3 mM glucose despite the time-average inhibition of glucagon release. Illustration based on data from Hellman et al. 2009 (57).

There are obviously reasons to doubt that inhibitory β-cell factors mediate glucose regulation of glucagon in hypoglycaemia. However, the involvement of these factors in direct control of glucagon secretion in hyperglycaemia is also questionable. Studies of human (57) and mouse (58) islets exposed to 20 mM glucose have revealed that insulin and glucagon are released in pulses that are synchronized in opposite phase (Figure 3). Such synchronization apparently explains why the circulating concentrations of the two hormones also oscillate in opposite phase in healthy human subjects (59). As mentioned above, Ca^2+^ is generally assumed to be the final trigger of both insulin and glucagon secretion, and pulsatile insulin release is indeed paralleled by synchronous β-cell oscillations of [Ca^2+^]_i_ (60). To mirror the secretory pattern of both hormones, [Ca^2+^]_i_ oscillations are consequently expected to synchronize in opposite phase between α- and β-cells within islets. Therefore, it was utterly surprising to find that the [Ca^2+^]_i_ oscillations of α- and β-cells tend to synchronize in the same phase in islets exposed to 20 mM glucose (36). Such synchronization seems to exclude that insulin, Zn^2+^ (45,49), or GABA (52) should mediate glucose inhibition of glucagon release by hyperpolarizing the α-cells to lower [Ca^2+^]_i_. A remaining possibility is that insulin instead acts by lowering cAMP (44), but another study did not find any effect of insulin receptor blockade on glucagon secretion at 20 mM glucose (36). Moreover, a putative action of GHB (53) remains to be explored.

### Stimulation by β-cell factors

A paracrine β-cell influence on the α-cells is necessarily not only inhibitory. The dose–response relationship for glucose-regulated glucagon release from mouse and human islets does not show monotonous inhibition. Maximal inhibition of glucagon release is obtained by raising glucose to 5–7 mM, and at higher concentrations the inhibition is gradually reduced (39–41). Concentrations above 20 mM even stimulate glucagon secretion from mouse islets (39). This U-shaped glucose concentration dependence indicates involvement of both inhibitory and stimulatory effects of glucose (Figure 1). The stimulatory component has been attributed to insulin based on the observation that this hormone stimulates rather than inhibits glucagon release from human islets exposed to 6 mM glucose (41), but studies with a receptor antagonist indicated that insulin is inhibitory (44) or lacks effect (36) on glucagon release under hyperglycaemic conditions. ATP from the insulin secretory granules and adenosine formed by extracellular ATP degradation are other putative amplifiers of glucagon secretion. The well-established stimulatory effect of adenosine is mediated by cAMP-elevating A_2_ receptors (50,61,62), whereas direct ATP actions on Ca^2+^-elevating P2Y_1_ purinoceptors have been claimed either to stimulate (51) or to inhibit (50) glucagon release. A particularly interesting aspect of P2Y_1_ purinoceptor signalling is that it can mediate synchronization of the insulin secretion-generating [Ca^2+^]_i_ oscillations among dispersed β-cells (63) and pancreatic islets (64) that lack physical contact. ATP released from the electrically coupled β-cells within an islet and acting on P2Y_1_ purinoceptors on non-coupled α-cells may well explain the above-mentioned paradoxical synchronization of Ca^2+^ oscillations between β- and α-cells (36). It seems possible that more than one inhibitory mechanism may be required to suppress glucagon secretion under hyperglycaemic conditions. General conclusions based on commonly used experimental conditions may consequently overemphasize paracrine β-cell factors in the direct control of glucagon release in general and during hypoglycaemia in particular. It is therefore reassuring that some recent studies focus on regulation of glucagon secretion in recovery from hypoglycaemia by studying the 0–7 mM glucose range (31,39,41,65).

### Inhibition by somatostatin from δ-cells

Somatostatin from the δ-cells is a potent inhibitor of both insulin and glucagon release and was early proposed as a paracrine regulator of glucagon release (66) mediating the inhibitory effect of hyperglycaemia (67). Preferential inhibition of glucagon release follows from the higher α- than β-cell sensitivity to somatostatin (68), probably reflecting somatostatin receptor (SSTR) subtype differences with SSTR2 dominating in rodent and human α-cells, and SSTR1 and SSTR5 in human and rodent β-cells, respectively (69–71). The closer spatial association between δ- and α-cells than that between δ- and β-cells in mouse islets (72) may also contribute to the preferential effect on glucagon secretion in this species. Somatostatin acts by reducing adenylyl cyclase production of cAMP via the Gα_i_ subunit coupled to the SSTR2 (44) and by de-priming the glucagon secretory granules after activation of the serine/threonine protein phosphatase calcineurin (73). Compared to β-cell factors, a conceptual advantage of somatostatin-mediated glucose inhibition of glucagon release is that it may operate also in hypoglycaemia, since glucose stimulates somatostatin release from mouse and human islets at similar concentrations that inhibit glucagon secretion (Figure 1) (39–41). Indeed, blockade of the α-cell SSTR2 (40), perturbation of Gα_i_ signal transduction by pertussis toxin treatment, or knockout of the somatostatin gene (65) enhances glucagon secretion from mouse islets exposed to 0, 1, or 7 mM glucose. However, these conditions do not prevent glucose elevation from inhibiting glucagon secretion. Pertussis toxin treatment also did not prevent inhibition of glucagon secretion from mouse islets in response to a 1 to 20 mM glucose increase (74), but in another study such treatment eliminated the inhibitory effect of a 1 to 11 mM glucose elevation by increasing secretion at the higher concentration (44). In human islets SSTR2 blockade had no effect on glucagon release at either 1 or 6 mM glucose (41), but in another study SSTR2 blockade prevented glucose inhibition by stimulating glucagon secretion at 11 mM but not at 1 mM glucose (44). On the other hand, pertussis toxin treatment eliminated glucose inhibition of glucagon release from human islets by inhibiting secretion at 1 mM but with no effect at 6 mM glucose (41). Taken together, these studies offer meagre support that somatostatin mediates glucose inhibition of glucagon release in the 0–7 mM range, and three studies concluded that the effect of somatostatin is tonic rather than regulatory (40,65,74).

The studies with SSTR2 blockade or pertussis toxin treatment provided conflicting results regarding involvement of somatostatin in glucose inhibition of glucagon release under hyperglycaemic conditions with 11 or 20 mM glucose (44,74). Tolbutamide, which like glucose acts by closing K_ATP_ channels to depolarize islet cells, stimulates the release of both insulin (25) and somatostatin (75) and inhibits glucagon release from islets exposed to 1 mM glucose (65). However, tolbutamide becomes stimulatory in pertussis toxin-treated islets, or in those from somatostatin knockout mice, and is also stimulatory when glucagon release is already inhibited by exposure to 7 mM glucose (65). Together with observations that glucose concentrations in the 0–20 mM range stimulate glucagon release from purified rat and mouse α-cells (76–78), these data indicate that the direct effect of K_ATP_ channel closure in α-cells is stimulatory (see below) and that this stimulation is counteracted by the simultaneously enhanced release of inhibitory somatostatin. Further evidence for a role of somatostatin in the regulation of glucagon secretion in hyperglycaemia is obtained from the kinetics of secretion. Pulsatile hormone release from human and mouse islets is generated by hyperglycaemia with coinciding pulses of insulin and somatostatin that are synchronized in opposite phase to the glucagon pulses (57,58). This relationship is consistent with the possibility that somatostatin peaks generate the nadirs of pulsatile glucagon release. Since pulsatile glucagon release is paradoxically synchronized in opposite phase to the α-cell oscillations of [Ca^2+^]_i_ (discussed above), such a role requires that somatostatin inhibits glucagon release when [Ca^2+^]_i_ is peaking. This may well be the case since SSTR2 blockade potently amplifies glucagon release from mouse islets exposed to 20 mM glucose without marked effects on the [Ca^2+^]_i_ oscillations in α-cells (36). Despite somatostatin’s documented potency as inhibitor of glucagon release (68) and the fact that its secretion increases concentration-dependently up to 30 mM glucose (Figure 1), its paracrine action does not prevent that inhibition of glucagon release from mouse and human islets is gradually diminished in the 7–20 mM range (40,41). The time-average rate of pulsatile glucagon secretion at 20 mM glucose actually represents a rather modest inhibition compared to secretion at 3 mM glucose (57,58).

## Juxtacrine control of glucagon release

Cells in direct contact often communicate by interactions between surface-bound ephrin and Eph molecules that function as ligands and receptors, or both, and can mediate bidirectional signalling to control cellular functions (79). Eph molecules are tyrosine kinase receptors activated by ephrin. ‘Forward’ signalling from β-cell ephrinA ligand to β-cell EphA receptors has been found to inhibit insulin release by promoting polymerization of an actin network beneath the plasma membrane, whereas ‘reverse’ signalling from EphA to ephrinA promotes secretion by actin depolymerization (80). Glucose amplification of forward signalling from β-cell ephrinA to α-cell EphA4 receptors was recently proposed to complement paracrine inhibition of glucagon release (Figure 2) and explain glucagon hypersecretion when β-cells disappear in diabetes (78). The most compelling argument for such ‘juxtacrine’ control of glucagon release is that artificial activation of forward signalling by exposure to IgG-fused ephrinA molecules restores characteristic glucose inhibition of glucagon release from purified α-cells, which otherwise respond with stimulated secretion (78). The proposed model with juxtacrine β-cell control of glucagon secretion from α-cells is interesting, but available data are limited and further studies are required to pinpoint whether the effects are regulatory or permissive. Apart from shedding light on the regulation of glucagon release, juxtacrine mechanisms may perhaps help to explain considerable differences in glucose-regulated [Ca^2+^]_i_ signalling between isolated α-cells (81) and those located in their natural environment within pancreatic islets (36).

## α-Cell intrinsic glucose control of glucagon release

According to the consensus model for glucose-induced insulin release (see above), metabolism of glucose is essential for β-cell recognition of the sugar as stimulus. Considering that hormone secretion is glucose-regulated also in islet α- and δ-cells and that these cell types originate from a common progenitor (82), it seems likely that glucose might be sensed by similar cellular mechanisms. Indeed, glucose metabolism has a central role in different models of α-cell-intrinsic glucose regulation of glucagon release. Glucose transport into rodent β-cells is mediated by the high-*K_m_* GLUT2 (83), which seems tailored for sensing blood glucose in hyperglycaemia. From this point of view it is natural that rodent α-cells, which are expected preferentially to sense glucose in hypoglycaemia, express the low-*K_m_* GLUT1 transporter (84,85). However, human β-cells that sense higher concentrations also express the low-*K_m_* GLUT1 (83), and glucose transport is not rate-limiting for its metabolism since it has been estimated to be 5- to 10-fold higher than glucose utilization in both α- and β-cells (84,85). The high-*K_m_* glucokinase, which is the dominating glucose-phosphorylating enzyme, is instead the rate-limiting glucosensor in β-cells (86) and may have this function also in α-cells with similar glucokinase activity (85). There is also some α-cell expression of low-*K_m_* hexokinase, but its significance is unclear, since this enzyme is saturated already by 1 mM glucose (85). The subsequent glycolytic flux is comparable in β- and α-cells (84), but glucose oxidation is considerably lower in α-cells (87,88) and the oxidative phosphorylation less efficient due to high expression of uncoupling protein 2. These differences are reflected by much smaller glucose-induced changes of ATP (36,47,89), FAD (90), and NAD(P)H (91) in α- than in β-cells. Glucose metabolism is nevertheless essential since a non-metabolizable glucose transport analogue has no effect, whereas glucokinase activation mimics glucose inhibition of glucagon release (65). If glucose metabolism in α- and β-cells controls glucagon and insulin release in hypo- and hyperglycaemia, respectively, it might be reflected by a relatively left-shifted dependence of metabolism on the glucose concentration in the α-cell. This seems to be the case since a 1 to 5 mM glucose elevation causes comparable ATP elevation in α- and β-cells, whereas the β-cell response is much greater after further elevation to 20 mM (36).

There are significant differences in the electrophysiology between β- and α-cells. In accordance with the secretory patterns the β-cells become electrically active and show [Ca^2+^]_i_ oscillations at high glucose, whereas the α-cells are active in the absence of the sugar. Glucose-induced closure of the K_ATP_ channels depolarizes the β-cells to open L-type Ca^2+^ channels that show half-maximal activation at –19 mV, and this Ca^2+^ permeability dominates the upstroke of the action potentials in the β-cell (25). It is more complex in α-cells with T-type Ca^2+^ channels that activate at potentials as low as –60 mV and tetrodotoxin (TTX)-sensitive Na^+^ channels that open at potentials more positive than –30 mV (28,29). There are also L-type and perhaps N-type Ca^2+^ channels in α-cells (30), although studies with more specific inhibitors indicated that the latter channels might be of P/Q-type (31). Whereas Ca^2+^ influx through the L-type channels triggers insulin release from β-cells, the relationship between Ca^2+^ influx into α-cells and glucagon release is more complicated. In rodent α-cells L-type channels dominate (80%) and mediate most Ca^2+^ influx, but their blockade has little effect on secretion. Conversely, blocking the non-L-type channels (20%) has modest effects on [Ca^2+^]_i_ but inhibits secretion to a similar extent as glucose elevation from 1 mM to 6 or 7 mM (30,31,92). The greater importance of the non-L-type channels is attributed to their close association with the glucagon-secretory granules (31,93). In the presence of adrenaline, which depolarizes α-cells, mobilizes Ca^2+^ from the endoplasmic reticulum (ER) (81,94), and elevates cAMP (33), entry of extracellular Ca^2+^ through the L-type channels triggers exocytosis of glucagon granules that do not co-localize with these channels (31,95). In human α-cells P/Q-type channels dominate over L-type channels (70%/20% of the integrated Ca^2+^ current) and account for most of the exocytosis, although they open very briefly and only mediate a fraction of the Ca^2+^ entry (96). The fact that glucose inhibition of glucagon release is associated with a rather modest reduction of [Ca^2+^]_i_ signalling (36,81,91,97,98) is consistent with the idea that Ca^2+^ channels close to the glucagon granules are most important. The background of additional Ca^2+^ entry may perhaps explain why glucose inhibition of glucagon release is far from complete and has a much lower dynamic range than glucose stimulation of insulin release (39–41).

### KATP channel-dependent glucose inhibition of glucagon release

**Figure 4.**
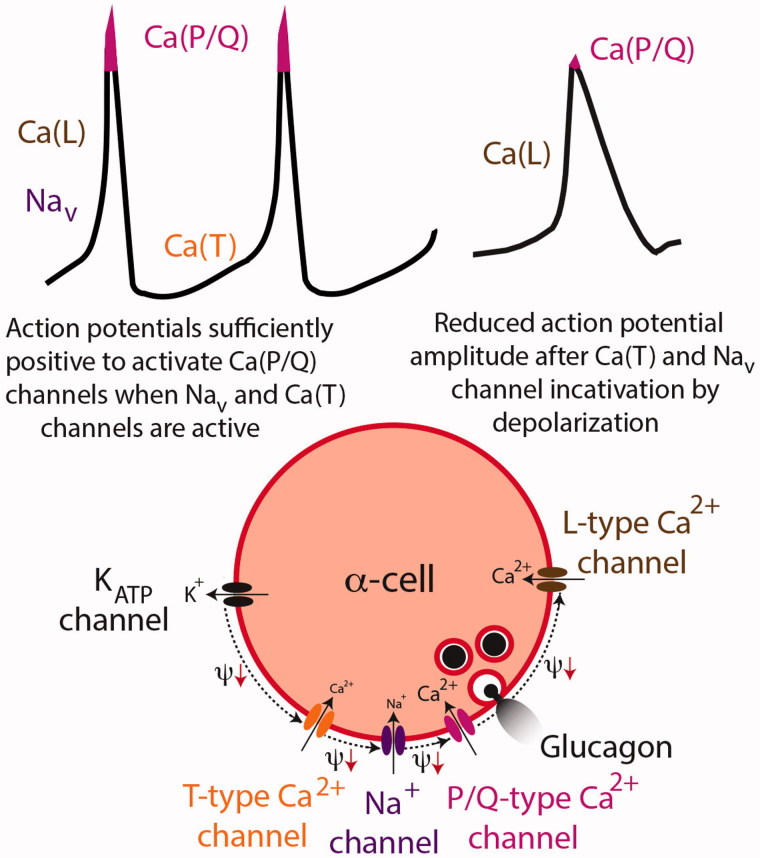
K_ATP_ channel-dependent regulation of glucagon secretion. In this model only a small fraction of the Ca^2+^ that enters the α-cells triggers glucagon release, and sustained depolarization inhibits secretion by reducing action potential amplitude. The upper left action potential illustration indicates the ion permeabilities that contribute to the different phases, and the lower panel shows these channels in an α-cell. Under stimulatory low-glucose conditions, the T-type Ca^2+^ channels fire spontaneously from about –60 mV and cause a depolarization (Ψ↓) that activates TTX-sensitive Na^+^ channels (Na_v_), which in turn activate L-type and briefly P/Q-type Ca^2+^ channels (pink peaks of the action potentials). The P/Q-type channels are more important for secretion than the L-type channels since the P/Q channels are located closer to the secretory granules. Repolarization depends on activation of voltage-dependent K^+^ channels (K_v_). Glucose inhibits glucagon secretion by slight sustained depolarization after closing the last few open K_ATP_ channels. The depolarization inactivates the T-type Ca^2+^ and Na_v_ channels, thereby preventing Ca^2+^ influx through the P/Q channels (upper right action potential) and glucagon release. Illustration based on Rorsman et al. 2012 (31).

According to the consensus hypothesis for glucose-stimulated insulin release, ATP acts by blocking K_ATP_ channels to depolarize the β-cell (see above). The most cited model for α-cell intrinsic regulation assumes the same sequence of events, meaning that glucose inhibits glucagon release by depolarizing the α-cells (31,41,93,99–101). The model is somewhat counterintuitive since it nevertheless incorporates the general acceptance that depolarization-induced influx of Ca^2+^ into the α-cells triggers glucagon secretion. The background to this riddle is that the α-cell K_ATP_ channel activity is almost maximally inhibited even in the absence of glucose (41) and that triggering of glucagon secretion by activation of high-threshold P/Q Ca^2+^ channels requires opening of low-threshold T-type Ca^2+^ and Na^+^ channels (28,91). ATP produced in response to glucose elevation closes the remaining K_ATP_ channels to depolarize the α-cells slightly and inactivate the low-threshold T-type Ca^2+^ and Na^+^ channels. In this situation the action potential amplitude is reduced and fails to activate secretion-triggering Ca^2+^ influx through the P/Q channels despite [Ca^2+^]_i_ elevation by activated L-channels (Figure 4). An implication of the model is that the action potentials that underlie glucagon secretion can only be generated in a narrow membrane potential window, sufficiently depolarized to activate the T-type Ca^2+^ channels and at the same time sufficiently hyperpolarized to prevent their inactivation as well as that of the TTX-sensitive Na^+^ channels (92). The presence of a narrow membrane potential window is supported by observations that slight depolarization by elevation of the K^+^ concentration mimics the inhibitory effect of glucose (100) and slight hyperpolarization with low concentrations of K_ATP_ channel-activating diazoxide can reverse glucose inhibition of glucagon secretion from batch-incubated islets (92,99), but such reversal could not be reproduced when measuring glucagon secretion from perifused islets (65). The observation that depolarization by complete K_ATP_ channel closure with tolbutamide inhibits glucagon release has also been taken to support the model (31,41). However, another study argued that increased release of somatostatin from the δ-cells mediates this inhibition, since tolbutamide strongly stimulated glucagon release from somatostatin knockout islets (65). The unexpected observation in normal mouse islets that a high concentration of tolbutamide enhances glucagon release, which has been inhibited by presence of 7 mM glucose, may reflect a direct tolbutamide stimulation of the α-cells that overwhelms indirect inhibition by somatostatin (65).

If the K_ATP_ channels are central for intrinsic α-cell recognition of glucose, channel knockout is expected to interfere with glucose regulation of glucagon release. Available data are conflicting also on this point. Whereas some studies found that the inhibitory effect of glucose is lost by K_ATP_ channel knockout (100,102), it was attenuated or essentially retained in other studies (65,103,104), and glucose inhibition of glucagon release was amplified when static inhibition of glucagon release by somatostatin was prevented by treating the K_ATP_ channel knockout islets with pertussis toxin (65). There are also divergent opinions on the effect of glucose after chemical knockout of the K_ATP_ channels by exposure to high concentrations of tolbutamide, which prevented glucose inhibition of glucagon release in one study (92) but not in another two (40,91). At high concentrations of diazoxide, which maximally open the K_ATP_ channels to hyperpolarize the α-cells and inhibit glucagon release, glucose can still inhibit secretion further (39,65).

There are very different estimates of the K_ATP_ channel expression in α-cells. *In situ* hybridization data indicated higher channel density than in β-cells (105), whereas expression was 7-fold lower in the α-cells based on binding of the fluorescently labelled sulfonylurea glibenclamide (106). In accordance with the latter estimation the whole-cell K_ATP_ channel conductance in α-cells is only about 10% of that in β-cells (28,99). Another difference between the two cell types is that the smaller α-cells have 100-fold lower membrane conductance (25,28), making the membrane potential more sensitive to small currents. From this point of view it is surprising that exposure to high concentrations of diazoxide to activate all K_ATP_ channels and hyperpolarize islet cells eliminates Ca^2+^ signalling in all β-cells but often fails to completely shut off [Ca^2+^]_i_ oscillations in α-cells (36,106). A possible interpretation is that α-cells have a more positive equilibrium potential for K^+^ and that hyperpolarization in response to K_ATP_ channel activation is insufficient for complete elimination of the action potentials that originate from membrane potentials of about –55 mV (28,29,93).

A fundamental aspect of the K_ATP_ channel hypothesis is that glucose depolarizes the α-cell, but it is, surprisingly, not settled how glucose affects the membrane potential. Diverging results have been obtained with invasive electrophysiological techniques with both depolarizing (93,100) and hyperpolarizing (28,105,107,108) effects of the sugar. The reason is probably that small stray currents associated with the invasive approach may significantly affect the membrane potential of the small α-cell with low membrane conductance (28). Inconsistent with the K_ATP_ channel hypothesis, membrane potential measurements with dye-based non-invasive technique indicate that glucose hyperpolarizes the α-cell (81,109).

### Alternative interpretations of data supporting the KATP channel-centred model

The K_ATP_ channel-centred model provides an explanation why slight α-cell hyperpolarization with low concentrations of diazoxide (92,99) and slight depolarization with small elevations of extracellular K^+^ (100) stimulates and inhibits glucagon release, respectively. Although such elegant clarifications of counterintuitive responses may seem convincing, alternative interpretations of these and some other experiments will be discussed. Considering that α-cells show surprisingly low sensitivity to high concentrations of diazoxide (see above), it seems possible that low concentrations may preferentially act on δ-cells to suppress somatostatin release and thus relieve some tonic inhibition of the α-cells. However, high concentrations of diazoxide likely inhibit glucagon release by hyperpolarizing the α-cells.

The Na^+^/K^+^-pumping ATPase is a major energy consumer in most types of cells and probably also in α-cells with much lower glucose-induced ATP production than β-cells (discussed above). Reduced Na^+^/K^+^ pumping may consequently underlie a more positive equilibrium potential for K^+^ in α- than in β-cells as speculated above. Moreover, if the energy utilization for ouabain-sensitive Na^+^/K^+^ pumping decreases with extracellular K^+^ elevation up to 14 mM like in squid axon (110), the inhibitory effect of small K^+^ elevations on glucagon release may be related to ATP elevation rather than to slight depolarization. This interpretation is consistent with a central role of metabolism in glucose-inhibited glucagon release without necessarily involving K_ATP_ channels.

Since the α-cell K_ATP_ channel activity is almost maximally inhibited in the absence of glucose (41), it is difficult to understand how low and high concentrations of tolbutamide can stimulate (92) and inhibit (31,41,92) glucagon release, respectively, if α-cell K_ATP_ channels were the sole target. The K_ATP_ channel-centred model offers no explanation for the stimulatory effect of low concentrations of tolbutamide (92). Stimulation is consistent with reports of α-cell depolarization with [Ca^2+^]_i_ elevation after K_ATP_ channel closure by tolbutamide (40,81,91,111). However, in accordance with the K_ATP_ channel-centred model, it was instead the inhibitory effect of tolbutamide on glucagon release that was linked to α-cell depolarizing after K_ATP_ channel closure (31,41,92). As discussed above, another study attributed tolbutamide inhibition of glucagon release to stimulated somatostatin secretion, since a high concentration of tolbutamide strongly stimulated glucagon release from somatostatin knockout islets (65).

### Endoplasmic reticulum-dependent glucose inhibition of glucagon release

In most excitable cells depolarization underlies opening of voltage-dependent Ca^2+^ influx that triggers a cellular response. From this point of view one might expect that α-cells depolarize to release glucagon in the absence of glucose and that glucose elevation, contrary to the K_ATP_-centred model, inhibits secretion by α-cell hyperpolarization. Although opinions differ about the glucose effect on α-cell membrane potential (see above), hyperpolarization seems to be the most common observation (28,81,105,107–109). The K_ATP_ channel-centred model is based on a special ion channel repertoire in α-cells that differs from that in β-cells, but the involvement of these channels in action potential generation does not exclude that glucose inhibits glucagon release by hyperpolarizing α-cells. In β-cells, glucose elevation rapidly generates ATP (112) and slight depolarization (113) that coincides with lowering of [Ca^2+^]_i_ (113,114), since ATP not only blocks K_ATP_ channels but also energizes the sarco(endo)plasmic reticulum Ca^2+^ ATPase (SERCA-pump) to sequester Ca^2+^ in the endoplasmic reticulum (ER). It is not until the depolarization is sufficient to open voltage-dependent Ca^2+^ channels that [Ca^2+^]_i_ increases (113) to trigger first-phase insulin secretion (115). Similar glucose-induced lowering of [Ca^2+^]_i_ by sequestration in the ER was early observed in guinea pig α-cells (116). Adrenaline, which in α-cells acts on α_1_- and β-adrenergic receptors to generate inositol (1,4,5)-trisphosphate (IP_3_) and cAMP, respectively (94), was found to raise [Ca^2+^]_i_ by mobilizing the glucose-incorporated Ca^2+^ (116). The opposite effects on [Ca^2+^]_i_ were therefore proposed to explain glucose inhibition and adrenaline stimulation of glucagon release (116). While this may seem to make sense, it is unlikely that ER sequestration of Ca^2+^ explains sustained inhibition of glucagon release, since the Ca^2+^ storage capacity is limited. However, the Ca^2+^ filling of the ER controls a store-operated depolarizing current in the plasma membrane that is at least partly carried by Ca^2+^ influx. In β- and α-cells the store-operated current is small, and alone it only causes modest [Ca^2+^]_i_ elevation (81,117,118). Activation of the store-operated current by SERCA inhibition marginally elevates basal [Ca^2+^]_i_ in β-cells (113), whereas α-cells react with a pronounced [Ca^2+^]_i_ elevation involving also voltage-dependent Ca^2+^ entry (81) that stimulates glucagon release (40) and prevents (40) or reduces (100) inhibition by glucose. The likely reason for this difference is that the low membrane conductance of α-cells (28) makes the membrane potential sufficiently sensitive to small depolarizing currents that activate voltage-dependent Ca^2+^ influx. The high sensitivity to small currents is the basis for glucagon release control by store-operated glucose sensing in α-cells (40,81).

**Figure 5.**
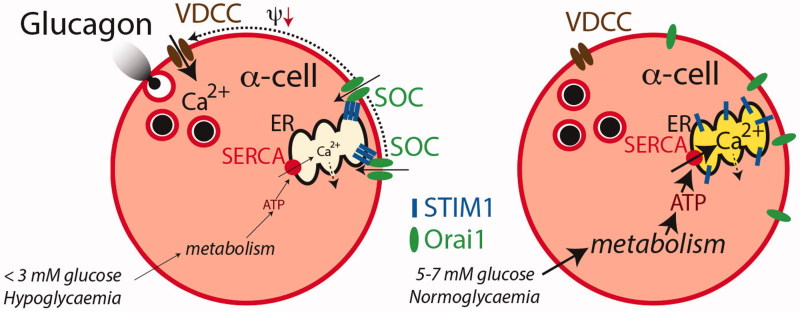
Endoplasmic reticulum-dependent regulation of glucagon release. Under hypoglycaemic conditions (left) the SERCA pump is poorly energized and Ca^2+^ leakage from the ER (dotted arrow) exceeds accumulation (solid arrow). Ca^2+^ depletion of the ER is associated with STIM1 aggregation and localization to the near plasma membrane where it co-clusters with plasma membrane Orai1 molecules that form store-operated cation channels (SOC). The resulting cation influx depolarizes (Ψ↓) the α-cell and opens voltage-dependent Ca^2+^ channels (VDCC). The subsequent influx of Ca^2+^ triggers glucagon release. In normoglycaemia the SERCA pump is properly energized and Ca^2+^ accumulation exceeds leakage to fill the ER. The STIM1 molecules then de-aggregate and translocate to the bulk ER and the SOCs disintegrate into Orai1 molecules thus shutting off the depolarizing store-operated cation influx and Ca^2+^ entry through VDCC as well as glucagon release. Illustration based on Liu et al. 2004 (81), Vieira et al. 2007 (40), and Tian et al. 2012 (122).

The store-operated Ca^2+^ influx in β-cells is inversely related to the Ca^2+^ content of the ER in a graded manner (118). Store-operated control of glucagon release from the α-cells implies that under hypoglycaemia, with little ATP to fuel the SERCA pump, there will be a net release of Ca^2+^ from the ER and activation of the depolarizing store-operated pathway, resulting in voltage-dependent Ca^2+^ influx and glucagon release (Figure 5). During return to normoglycaemia the SERCA pump is gradually more energized to fill the ER with Ca^2+^ and shut off the stimulatory cascade (40,81). Some important differences between α- and β-cells provide arguments in support of the store-operated mechanism. In β-cells 8–20 mM glucose is required to fill the ER (119–121), whereas maximum is reached at 3 mM in the α-cells (81). A similar difference is apparent when comparing the spatio-temporal kinetics of the ER Ca^2+^ sensor STIM1 and the plasma membrane Ca^2+^ channel protein Orai1, which are molecular components of the store-operated pathway. Emptying of ER Ca^2+^ induces STIM1 translocation from the ER to the plasma membrane where it co-clusters with Orai1 to activate the store-operated current in both α- and β-cells, and glucose reverses this process by stimulating re-translocation of STIM1 to the ER in a graded fashion, thus shutting off the store-operated current (122). In β-cells the re-translocation is saturated by 11–20 mM glucose, whereas only 3 mM is required in α-cells. Similar hypoglycaemia-like glucose concentrations consequently control glucagon release and the store-operated pathway in α-cells, whereas quite different concentrations control this pathway in β-cells. The simple idea that glucose inhibition and adrenaline stimulation of glucagon release are explained by stimulation of ER Ca^2+^ sequestration and release, respectively (116), obviously makes sense when the store-operated pathway is taken into account.

### Other α-cell mechanisms for glucose-inhibited glucagon release

Like in store-operated control of glucagon release, another two hypotheses assume that glucose inhibits secretion by hyperpolarizing the α-cells to shut off voltage-dependent Ca^2+^ entry. Glucose-generated ATP was thus proposed to act by energizing the Na^+^/K^+^ pump (123). In most types of cells the electrogenic effect of this pump is small (124) but may be significant in α-cells due to the their low membrane conductance (28). The other hypothesis is based on the observation that glucose elevations inhibiting glucagon secretion cause α-cell swelling (125) and activation of volume-regulated channels, leading to hyperpolarizing Cl^-^ influx (126,127). Another α-cell-intrinsic model is independent of the membrane potential and changes in [Ca^2+^]_i_ and focuses on the reduction of AMP that accompanies glucose-induced ATP elevation. The lowering of AMP is assumed to inhibit glucagon release by inactivating AMP-dependent protein kinase (AMPK) (34). Accordingly, activation of AMPK was found to stimulate glucagon release from a clonal α-cell line and mouse pancreatic islets, whereas a dominant-negative form of the kinase blocked the stimulatory effects of low glucose (34). However, knockout of the catalytic α1 sub-unit of AMPK instead sensitized glucagon secretion to the inhibitory action of glucose (128). Currently available data on the involvement of AMPK in glucose-regulated glucagon secretion therefore seem somewhat contradictory.

### Glucose stimulation of glucagon release

Arguments that glucose inhibition of glucagon release from pancreatic islets depends on para- (76,77) and/or juxtacrine mechanisms (78) were obtained from observations that glucose instead stimulates glucagon release from purified α-cells. A major difference between isolated α-cells and those located within islets is that the latter show consistent [Ca^2+^]_i_ activity with oscillations at 1–3 mM glucose (36,77), whereas such activity is observed in less than 10% of isolated α-cells (81). Intra-islet location apparently stimulates the α-cells to induce [Ca^2+^]_i_ activity and glucagon release, which is a likely requirement for observing inhibition by glucose. The stimulation may involve autocrine α-cell factors (see above) that act in the narrow intercellular space of islets but whose effect is lost by dilution when studying purified or isolated α-cells. Indeed, increasing glucose in the 1–20 mM range concentration-dependently inhibits [Ca^2+^]_i_ signalling in isolated α-cells, provided that they are already depolarized (40), which resembles the temporary inhibition of [Ca^2+^]_i_ signalling observed in islet-located α-cells after a 3–20 mM glucose elevation (36). Glucose-induced stimulation of glucagon release from purified α-cells is supposed to mirror that in β-cells with voltage-dependent [Ca^2+^]_i_ elevation due to depolarization by closure of K_ATP_ channels (76), which is opposite to the hyperpolarizing effect of glucose observed in most studies of isolated α-cells (28,81,105,107) or those located *in situ* within the islets (108,109). Also, glucagon release from pancreatic islets shows a stimulatory glucose component, since maximal inhibition at 5–7 mM is followed by a gradually reduced inhibition at higher concentrations (39–41) that transforms into stimulation above 20 mM of the sugar (Figure 1) (39). Even if closure of the K_ATP_ channels were involved in the stimulatory effect of glucose on glucagon secretion from purified α-cells it seems unlikely that this mechanism accounts for stimulation by hyperglycaemic concentrations of the sugar, since the α-cell K_ATP_ channel activity is almost maximally inhibited already in the absence of glucose (41). The question arises whether another process than [Ca^2+^]_i_ elevation may explain glucose stimulation. Since glucose inhibition of glucagon release is associated with a rather modest reduction of [Ca^2+^]_i_ signalling (36,81,91,97,98), it is possible that secretion shows a greater dependence on amplifying factors and that Ca^2+^ has a more permissive role. Glucose-induced elevation of cAMP in α-cells has thus been suggested to underlie glucose stimulation of glucagon release (33), but available data on α-cell cAMP are so far limited.

## Conclusions

**Figure 6.**
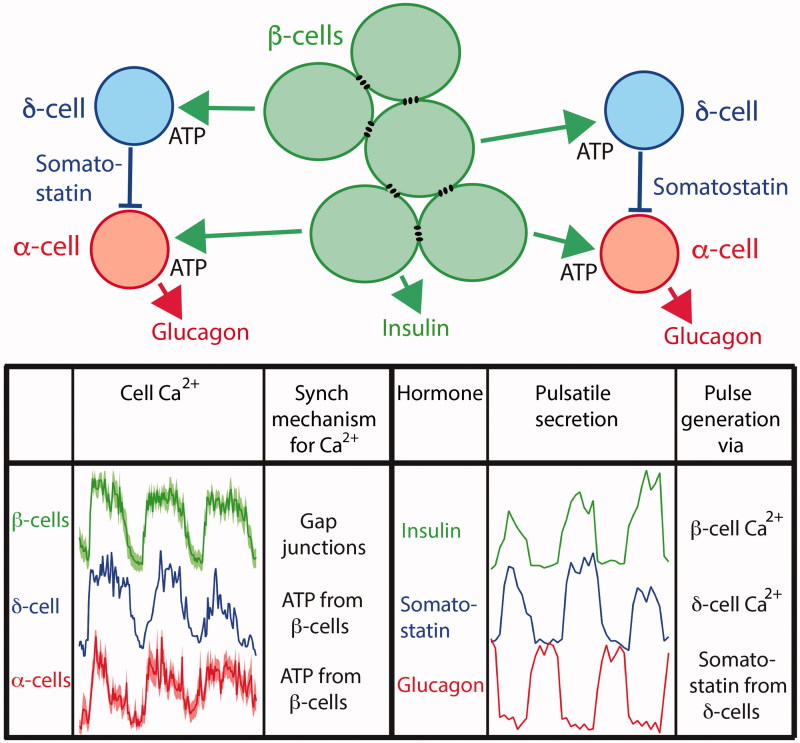
Model for paracrine generation of pulsatile glucagon release in hyperglycaemia. The gap junction-coupled β-cells synchronize their Ca^2+^ oscillations with those of the non-coupled δ-cells and α-cells by releasing factors like ATP. Therefore, the Ca^2+^ oscillations in the δ-cells will generate pulsatile release of somatostatin in phase with that of insulin. Since somatostatin potently inhibits glucagon release with little effect on α-cell Ca^2+^, this inhibition will generate pulsatile glucagon release in opposite phase to insulin and somatostatin pulsatility. The lower left graphs show oscillations of the sub-plasma membrane Ca^2+^ concentration means (dark colour) ± SEM (light colour) of 6 β- and 13 α-cells within a single mouse islet (modified from Li et al. 2015 (36)), as well as an adapted recording from a δ-cell based on preliminary experiments. The graphs showing pulsatile secretion (lower right) are based on data from Hellman et al. 2009 (57).

A reason why glucose regulation of glucagon secretion is poorly understood is probably that experimental efforts have mostly been focused on finding a single mechanism. It has become increasingly evident that glucose has both inhibitory and stimulatory effects that act directly on the α-cell or indirectly via paracrine and/or juxtacrine signalling from other islet cell types. A further complication is that these mechanisms contribute differently depending on the prevailing glucose concentration. Under hypoglycaemic conditions α-cell-intrinsic glucose sensing and regulation of glucagon release is probably dominating. Although the two major hypotheses for such sensing seem incompatible in predicting that glucose inhibits glucagon secretion by either depolarizing (31,41,93,99,100) or hyperpolarizing (40,81) the α-cell, respectively, a model has been proposed that combines these mechanisms to explain the non-monotonic dependence of glucagon secretion on glucose (129). Most models for α-cell-intrinsic glucose regulation of glucagon release focus on Ca^2+^ as the trigger of secretion. However, one should also consider the possibility that Ca^2+^ has a more permissive role and that α-cell-intrinsic glucose regulation in terms of inhibition and stimulation is mediated by modulating factors like cAMP and perhaps diacylglycerol. This alternative is particularly attractive considering that glucose only modestly decreases α-cell [Ca^2+^]_i_ signalling, which likely explains why even maximally inhibited glucagon secretion is only reduced to 25%–45% of maximal release (40,41). In hyperglycaemia paracrine factors become more important for glucose regulation of glucagon release, and the stimulated β-cells ultimately generate pulsatile secretion of the other islet hormones (Figure 6). Due to gap junction coupling they release insulin together with synchronizing paracrine factors like ATP in a co-ordinated pulsatile fashion to entrain the [Ca^2+^]_i_ oscillation of the non-coupled δ- and α-cells into a common islet phase. The [Ca^2+^]_i_ entrainment of β- and δ-cells likely explains the coinciding pulses of insulin and somatostatin release. However, the somatostatin pulses inhibit glucagon release despite peaks of α-cell [Ca^2+^]_i_, thus clarifying how pulsatile glucagon release is synchronized in opposite phase α-cell [Ca^2+^]_i_ as well as to pulsatile release of the other hormones. Since the stimulatory component of glucose on glucagon secretion increases with sugar concentration, multiple inhibitory processes may be required to ascertain dominating inhibition of glucagon secretion. Glucagon release in diabetes shows two distinct abnormalities—hypersecretion during hyperglycaemia and a failing secretory response to hypoglycaemia. Both abnormalities might be explained if the stimulatory glucose component is somehow amplified to dominate over the inhibitory mechanisms. The molecular events that underlie glucose stimulation of glucagon release should therefore be identified and explored as potential targets in diabetes therapy.

## Disclosure statement

The author reports no conflicts of interest.

## Funding information

This work was supported by grants from the Swedish Research Council (55X-06240) and the Swedish Diabetes Association (DIA2014-071, DIA2015-027).

## References

[1] Langerhans P. Beiträge zur mikroskopischen Anatomie der Bauchspeicheldrüse Berlin: Gustav Lange; 1869.

[2] Diamare V. Studii comperativi sulle isole di Langerhans del pancreas. Internat Monatsch Anat Physiol. 1899;16:155–209.

[3] Lane MA. The cytological characters of the areas of Langerhans. Am J Anat. 1907;7:409–22.

[4] Homans J. A study of experimental diabetes in the canine and its relation to human diabetes. J Med Res. 1915;33:1–53. 19972253 PMC2094499

[5] Banting FG, Best CH. The internal secretion of the pancreas. J Lab Clin Med. 1922;7:251–66. 17582843

[6] Murlin JR, Clough HD, Gibbs CBF, Stokes AM. Aqueous extracts of pancreas I. Influence on the carbohydrate metabolism of depancreatized animals. J Biol Chem. 1923;56:253–96.

[7] Kimball C, Murlin J. Aqueous extracts of pancreas III. Some precipitation reactions of insulin. J Biol Chem. 1923;58:337–46.

[8] Sutherland EW, De Duve C. Origin and distribution of the hyperglycemic-glycogenolytic factor of the pancreas. J Biol Chem. 1948;175:663–74. 18880761

[9] Hellerström C, Hellman B. Some aspects of silver impregnation of the islets of Langerhans in the rat. Acta Endocrinol (Copenh). 1960;35:518–32. 13713144 10.1530/acta.0.xxxv0518

[10] Hellerström C, Hellman B. Reactions of the two types of A cells in the islets of Langerhans after administration of glucagon. Acta Endocrinol (Copenh). 1962;41:116–22. 13906260 10.1530/acta.0.0410116

[11] Hellerström C, Howell SL, Edwards JC, Andersson A. An investigation of glucagon biosynthesis in isolated pancreatic islets of guinea pigs. FEBS Lett. 1972;27:97–101. 11946816 10.1016/0014-5793(72)80418-6

[12] Andersson A, Petersson B, Westman J, Edwards J, Lundqvist G, Hellerström C. Tissue culture of A_2_ cell-rich pancreatic islets isolated from guinea-pigs. J Cell Biol. 1973;57:241–7. 4266245 10.1083/jcb.57.1.241 PMC2108945

[13] Bloom W. A new type of granular cell in the islets of Langerhans of man. Anat Rec. 1931;49:363–70.

[14] Hellman B, Lernmark A. Inhibition of the in vitro secretion of insulin by an extract of pancreatic α_1_ cells. Endocrinology. 1969;84:1484–8. 4889114 10.1210/endo-84-6-1484

[15] Goldsmith PC, Rose JC, Arimura A, Ganong WF. Ultrastructural localization of somatostatin in pancreatic islets of the rat. Endocrinology. 1975;97:1061–4. 1104349 10.1210/endo-97-4-1061

[16] Banting FG, Best CH, Collip JB, Campbell WR, Fletcher AA. Pancreatic extracts in the treatment of diabetes mellitus. Can Med Assoc J. 1922;12:141–6. 20314060 PMC1524425

[17] Cryer PE. Minireview: glucagon in the pathogenesis of hypoglycemia and hyperglycemia in diabetes. Endocrinology. 2012;153:1039–48. 22166985 10.1210/en.2011-1499 PMC3281526

[18] Unger RH, Cherrington AD. Glucagonocentric restructuring of diabetes: a pathophysiologic and therapeutic makeover. J Clin Invest. 2012;122:4–12. 22214853 10.1172/JCI60016 PMC3248306

[19] Wang MY, Yan H, Shi Z, Evans MR, Yu X, Lee Y, et al Glucagon receptor antibody completely suppresses type 1 diabetes phenotype without insulin by disrupting a novel diabetogenic pathway. Proc Natl Acad Sci U S A. 2015;112:2503–8. 25675519 10.1073/pnas.1424934112 PMC4345619

[20] Steenberg VR, Jensen SM, Pedersen J, Madsen AN, Windeløv JA, Holst B, et al Acute disruption of glucagon secretion or action does not improve glucose tolerance in an insulin-deficient mouse model of diabetes. Diabetologia. 2016;59:363–70. 26537124 10.1007/s00125-015-3794-2

[21] Gerich JE. Glucose counterregulation and its impact on diabetes mellitus. Diabetes. 1988;37:1608–17. 3056759 10.2337/diab.37.12.1608

[22] Thorens B. Brain glucose sensing and neural regulation of insulin and glucagon secretion. Diabetes Obes Metab. 2011;13(Suppl 1):82–8. 21824260 10.1111/j.1463-1326.2011.01453.x

[23] Thorens B. Neural regulation of pancreatic islet cell mass and function. Diabetes Obes Metab. 2014;16(Suppl1):87–95. 25200301 10.1111/dom.12346

[24] Rodriguez-Diaz R, Dando R, Jacques-Silva MC, Fachado A, Molina J, Abdulreda MH, et al Alpha cells secrete acetylcholine as a non-neuronal paracrine signal priming beta cell function in humans. Nat Med. 2011;17:888–92. 21685896 10.1038/nm.2371 PMC3132226

[25] Ashcroft FM, Rorsman P. Electrophysiology of the pancreatic β-cell. Prog Biophys Mol Biol. 1989;54:87–143. 2484976 10.1016/0079-6107(89)90013-8

[26] Tengholm A, Gylfe E. Oscillatory control of insulin secretion. Mol Cell Endocrinol. 2009;297:58–72. 18706473 10.1016/j.mce.2008.07.009

[27] Gylfe E, Tengholm A. Neurotransmitter control of islet hormone pulsatility. Diabetes Obes Metab. 2014;16(Suppl 1):102–10. 25200303 10.1111/dom.12345

[28] Barg S, Galvanovskis J, Göpel SO, Rorsman P, Eliasson L. Tight coupling between electrical activity and exocytosis in mouse glucagon-secreting α-cells. Diabetes. 2000;49:1500–10. 10969834 10.2337/diabetes.49.9.1500

[29] Huang YC, Rupnik MS, Karimian N, Herrera PL, Gilon P, Feng ZP, et al In situ electrophysiological examination of pancreatic α cells in the streptozotocin-induced diabetes model, revealing the cellular basis of glucagon hypersecretion. Diabetes. 2013;62:519–30. 23043159 10.2337/db11-0786 PMC3554363

[30] Gromada J, Bokvist K, Ding WG, Barg S, Buschard K, Renström E, et al Adrenaline stimulates glucagon secretion in pancreatic A-cells by increasing the Ca^2+^ current and the number of granules close to the L-type Ca^2+^ channels. J Gen Physiol. 1997;110:217–28. 9276750 10.1085/jgp.110.3.217 PMC2229364

[31] Rorsman P, Braun M, Zhang Q. Regulation of calcium in pancreatic α- and β-cells in health and disease. Cell Calcium. 2012;51:300–8. 22177710 10.1016/j.ceca.2011.11.006 PMC3334273

[32] Ma X, Zhang Y, Gromada J, Sewing S, Berggren PO, Buschard K, et al Glucagon stimulates exocytosis in mouse and rat pancreatic α-cells by binding to glucagon receptors. Mol Endocrinol. 2005;19:198–212. 15459251 10.1210/me.2004-0059

[33] Tian G, Sandler S, Gylfe E, Tengholm A. Glucose- and hormone-induced cAMP oscillations in α- and β-cells within intact pancreatic islets. Diabetes. 2011;60:1535–43. 21444924 10.2337/db10-1087 PMC3292328

[34] Leclerc I, Sun G, Morris C, Fernandez-Millan E, Nyirenda M, Rutter GA. AMP-activated protein kinase regulates glucagon secretion from mouse pancreatic alpha cells. Diabetologia. 2011;54:125–34. 20938634 10.1007/s00125-010-1929-z PMC6101198

[35] Le Marchand SJ, Piston DW. Glucose decouples intracellular Ca^2+^ activity from glucagon secretion in mouse pancreatic islet alpha-cells. PLoS One. 2012;7:e47084. 23077547 10.1371/journal.pone.0047084 PMC3471958

[36] Li J, Yu Q, Ahooghalandari P, Gribble FM, Reimann F, Tengholm A, et al Sub-membrane ATP and Ca^2+^ kinetics in α-cells – unexpected signalling for glucagon secretion. FASEB J. 2015;29:3379–88. 25911612 10.1096/fj.14-265918 PMC4539996

[37] Gylfe E, Gilon P. Glucose regulation of glucagon secretion. Diabetes Res Clin Pract. 2014;103:1–10. 24367972 10.1016/j.diabres.2013.11.019

[38] Gromada J, Franklin I, Wollheim CB. α-Cells of the endocrine pancreas: 35 years of research but the enigma remains. Endocr Rev. 2007;28:84–116. 17261637 10.1210/er.2006-0007

[39] Salehi A, Vieira E, Gylfe E. Paradoxical stimulation of glucagon secretion by high glucose concentrations. Diabetes. 2006;55:2318–23. 16873696 10.2337/db06-0080

[40] Vieira E, Salehi A, Gylfe E. Glucose inhibits glucagon secretion by a direct effect on mouse pancreatic α-cells. Diabetologia. 2007;50:370–9. 17136393 10.1007/s00125-006-0511-1

[41] Walker JN, Ramracheya R, Zhang Q, Johnson PR, Braun M, Rorsman P. Regulation of glucagon secretion by glucose: paracrine, intrinsic or both? Diabetes Obes Metab. 2011;13(Suppl 1):95–105. 21824262 10.1111/j.1463-1326.2011.01450.x

[42] Cabrera O, Jacques-Silva MC, Speier S, Yang SN, Kohler M, Fachado A, et al Glutamate is a positive autocrine signal for glucagon release. Cell Metab. 2008;7:545–54. 18522835 10.1016/j.cmet.2008.03.004 PMC4396785

[43] Östenson CG. Regulation of glucagon release: effects of insulin on the pancreatic A_2_-cell of the guinea pig. Diabetologia. 1979;17:325–30. 387506 10.1007/BF01235889

[44] Elliott AD, Ustione A, Piston DW. Somatostatin and insulin mediate glucose-inhibited glucagon secretion in the pancreatic α-cell by lowering cAMP. Am J Physiol Endocrinol Metab. 2015;308:E130–43. 25406263 10.1152/ajpendo.00344.2014 PMC4297778

[45] Franklin I, Gromada J, Gjinovci A, Theander S, Wollheim CB. β-Cell secretory products activate α-cell ATP-dependent potassium channels to inhibit glucagon release. Diabetes. 2005;54:1808–15. 15919803 10.2337/diabetes.54.6.1808

[46] Xu E, Kumar M, Zhang Y, Ju W, Obata T, Zhang N, et al Intra-islet insulin suppresses glucagon release via GABA-GABA_A_ receptor system. Cell Metab. 2006;3:47–58. 16399504 10.1016/j.cmet.2005.11.015

[47] Ishihara H, Maechler P, Gjinovci A, Herrera PL, Wollheim CB. Islet β-cell secretion determines glucagon release from neighbouring α-cells. Nat Cell Biol. 2003;5:330–5. 12640462 10.1038/ncb951

[48] Zhou H, Zhang T, Harmon JS, Bryan J, Robertson RP. Zinc, not insulin, regulates the rat α-cell response to hypoglycemia in vivo. Diabetes. 2007;56:1107–12. 17317764 10.2337/db06-1454

[49] Slucca M, Harmon JS, Oseid EA, Bryan J, Robertson RP. ATP-sensitive K^+^ channel mediates the zinc switch-off signal for glucagon response during glucose deprivation. Diabetes. 2010;59:128–34. 19808893 10.2337/db09-1098 PMC2797913

[50] Tuduri E, Filiputti E, Carneiro EM, Quesada I. Inhibition of Ca^2+^ signaling and glucagon secretion in mouse pancreatic α-cells by extracellular ATP and purinergic receptors. Am J Physiol Endocrinol Metab. 2008;294:E952–60. 18349114 10.1152/ajpendo.00641.2007

[51] Grapengiesser E, Salehi A, Qader SS, Hellman B. Glucose induces glucagon release pulses antisynchronous with insulin and sensitive to purinoceptor inhibition. Endocrinology. 2006;147:3472–7. 16614082 10.1210/en.2005-1431

[52] Rorsman P, Berggren PO, Bokvist K, Ericson H, Möhler H, Östenson CG, et al Glucose-inhibition of glucagon secretion involves activation of GABA_A_-receptor chloride channels. Nature. 1989;341:233–6. 2550826 10.1038/341233a0

[53] Li C, Liu C, Nissim I, Chen J, Chen P, Doliba N, et al Regulation of glucagon secretion in normal and diabetic human islets by γ-hydroxybutyrate and glycine. J Biol Chem. 2013;288:3938–51. 23266825 10.1074/jbc.M112.385682 PMC3567647

[54] Braun M, Wendt A, Birnir B, Broman J, Eliasson L, Galvanovskis J, et al Regulated exocytosis of GABA-containing synaptic-like microvesicles in pancreatic β-cells. J Gen Physiol. 2004;123:191–204. 14769845 10.1085/jgp.200308966 PMC2217446

[55] Braun M, Wendt A, Karanauskaite J, Galvanovskis J, Clark A, MacDonald PE, et al Corelease and differential exit via the fusion pore of GABA, serotonin, and ATP from LDCV in rat pancreatic β cells. J Gen Physiol. 2007;129:221–31. 17296927 10.1085/jgp.200609658 PMC2151613

[56] Pizarro-Delgado J, Braun M, Hernandez-Fisac I, Martin-Del-Rio R, Tamarit-Rodriguez J. Glucose promotion of GABA metabolism contributes to the stimulation of insulin secretion in β-cells. Biochem J. 2010;431:381–9. 20695849 10.1042/BJ20100714

[57] Hellman B, Salehi A, Gylfe E, Dansk H, Grapengiesser E. Glucose generates coincident insulin and somatostatin pulses and anti-synchronous glucagon pulses from human pancreatic islets. Endocrinology. 2009;150:5334–40. 19819962 10.1210/en.2009-0600

[58] Hellman B, Salehi A, Grapengiesser E, Gylfe E. Isolated mouse islets respond to glucose with an initial peak of glucagon release followed by pulses of insulin and somatostatin in antisynchrony with glucagon. Biochem Biophys Res Commun. 2012;417:1219–23. 22227186 10.1016/j.bbrc.2011.12.113

[59] Rohrer S, Menge BA, Gruber L, Deacon CF, Schmidt WE, Veldhuis JD, et al Impaired crosstalk between pulsatile insulin and glucagon secretion in prediabetic individuals. J Clin Endocrinol Metab. 2012;97:E791–5. 22399501 10.1210/jc.2011-3439

[60] Bergsten P, Grapengiesser E, Gylfe E, Tengholm A, Hellman B. Synchronous oscillations of cytoplasmic Ca^2+^ and insulin release in glucose-stimulated pancreatic islets. J Biol Chem. 1994;269:8749–53. 8132606

[61] Chapal J, Loubatieres-Mariani MM, Petit P, Roye M. Evidence for an A2-subtype adenosine receptor on pancreatic glucagon secreting cells. Br J Pharmacol. 1985;86:565–9. 2998522 10.1111/j.1476-5381.1985.tb08932.x PMC1916740

[62] Petit P, Loubatieres-Mariani MM, Keppens S, Sheehan MJ. Purinergic receptors and metabolic function. Drug Dev Res. 1996;39:413–25.

[63] Grapengiesser E, Dansk H, Hellman B. Pulses of external ATP aid to the synchronization of pancreatic β-cells by generating premature Ca^2+^ oscillations. Biochem Pharmacol. 2004;68:667–74. 15276074 10.1016/j.bcp.2004.04.018

[64] Gylfe E, Grapengiesser E, Dansk H, Hellman B. The neurotransmitter ATP triggers Ca^2+^ responses promoting coordination of pancreatic islet oscillations. Pancreas. 2012;41:258–63. 22076565 10.1097/MPA.0b013e3182240586

[65] Cheng-Xue R, Gómez-Ruiz A, Antoine N, Noêl LA, Chae HY, Ravier MA, et al Tolbutamide controls glucagon release from mouse islets differently than glucose: involvement of K_ATP_ channels from both α- and β-cells. Diabetes. 2013;62:1612–22. 23382449 10.2337/db12-0347 PMC3636641

[66] Starke A, Imamura T, Unger RH. Relationship of glucagon suppression by insulin and somatostatin to the ambient glucose concentration. J Clin Invest. 1987;79:20–4. 2878938 10.1172/JCI112784 PMC423975

[67] Klaff LJ, Taborsky GJ Jr. Pancreatic somatostatin is a mediator of glucagon inhibition by hyperglycemia. Diabetes. 1987;36:592–6. 2883057 10.2337/diab.36.5.592

[68] Schuit FC, Derde MP, Pipeleers DG. Sensitivity of rat pancreatic A and B cells to somatostatin. Diabetologia. 1989;32:207–12. 2568961 10.1007/BF00265096

[69] Kumar U, Sasi R, Suresh S, Patel A, Thangaraju M, Metrakos P, et al Subtype-selective expression of the five somatostatin receptors (hSSTR1-5) in human pancreatic islet cells: a quantitative double-label immunohistochemical analysis. Diabetes. 1999;48:77–85. 9892225 10.2337/diabetes.48.1.77

[70] Strowski MZ, Parmar RM, Blake AD, Schaeffer JM. Somatostatin inhibits insulin and glucagon secretion via two receptors subtypes: an *in vitro* study of pancreatic islets from somatostatin receptor 2 knockout mice. Endocrinology. 2000;141:111–17. 10614629 10.1210/endo.141.1.7263

[71] Ludvigsen E, Carlsson C, Janson ET, Sandler S, Stridsberg M. Somatostatin receptor 1-5; expression profiles during rat development. Ups J Med Sci. 2015;120:157–68. 25926390 10.3109/03009734.2015.1035413 PMC4526871

[72] Orci L, Unger RH. Functional subdivision of islets of Langerhans and possible role of D cells. Lancet. 1975;2:1243–4. 10.1016/s0140-6736(75)92078-4 53729

[73] Gromada J, Høy M, Buschard K, Salehi A, Rorsman P. Somatostatin inhibits exocytosis in rat pancreatic a-cells by G_i2_-dependent activation of calcineurin and depriming of secretory granules. J Physiol. 2001;535:519–32. 11533141 10.1111/j.1469-7793.2001.00519.x PMC2278803

[74] Göpel S, Zhang Q, Eliasson L, Ma XS, Galvanovskis J, Kanno T, et al Capacitance measurements of exocytosis in mouse pancreatic α-, β- and δ-cells within intact islets of Langerhans. J Physiol. 2004;556:711–26. 14966302 10.1113/jphysiol.2003.059675 PMC1664984

[75] Ipp E, Dobbs RE, Arimura A, Vale W, Harris V, Unger RH. Release of immunoreactive somatostatin from the pancreas in response to glucose, amino acids, pancreozymin-cholecystokinin and tolbutamide. J Clin Invest. 1977;60:760–5. 330567 10.1172/JCI108829 PMC372422

[76] Olsen HL, Theander S, Bokvist K, Buschard K, Wollheim CB, Gromada J. Glucose stimulates glucagon release in single rat α-cells by mechanisms that mirror the stimulus-secretion coupling in β-cells. Endocrinology. 2005;146:4861–70. 16081632 10.1210/en.2005-0800

[77] Le Marchand SJ, Piston DW. Glucose suppression of glucagon secretion: metabolic and calcium responses from α-cells in intact mouse pancreatic islets. J Biol Chem. 2010;285:14389–98. 20231269 10.1074/jbc.M109.069195 PMC2863245

[78] Hutchens T, Piston DW. EphA4 receptor forward signaling inhibits glucagon secretion from α-cells. Diabetes. 2015:3839–51. 26251403 10.2337/db15-0488 PMC4613968

[79] Miao H, Wang B. EphA receptor signaling–complexity and emerging themes. Semin Cell Dev Biol. 2012;23:16–25. 22040915 10.1016/j.semcdb.2011.10.013 PMC3374974

[80] Konstantinova I, Nikolova G, Ohara-Imaizumi M, Meda P, Kucera T, Zarbalis K, et al EphA-Ephrin-A-mediated β cell communication regulates insulin secretion from pancreatic islets. Cell. 2007;129:359–70. 17448994 10.1016/j.cell.2007.02.044

[81] Liu YJ, Vieira E, Gylfe E. A store-operated mechanism determines the activity of the electrically excitable glucagon-secreting pancreatic α-cell. Cell Calcium. 2004;35:357–65. 15036952 10.1016/j.ceca.2003.10.002

[82] Haumaitre C, Lenoir O, Scharfmann R. Directing cell differentiation with small-molecule histone deacetylase inhibitors: the example of promoting pancreatic endocrine cells. Cell Cycle. 2009;8:536–44. 19197155 10.4161/cc.8.4.7610

[83] De Vos A, Heimberg H, Quartier E, Huypens P, Bouwens L, Pipeleers D, et al Human and rat beta cells differ in glucose transporter but not in glucokinase gene expression. J Clin Invest. 1995;96:2489–95. 7593639 10.1172/JCI118308 PMC185903

[84] Heimberg H, De Vos A, Pipeleers DG, Thorens B, Schuit FC. Differences in glucose transporter gene expression between rat pancreatic α- and β-cells are correlated to differences in glucose transport but not in glucose utilization. J Biol Chem. 1995;270:8971–5. 7721807 10.1074/jbc.270.15.8971

[85] Heimberg H, De Vos A, Moens K, Quartier E, Bouwens L, Pipeleers D, et al The glucose sensor protein glucokinase is expressed in glucagon-producing α-cells. Proc Natl Acad Sci U S A. 1996;93:7036–41. 8692940 10.1073/pnas.93.14.7036 PMC38931

[86] Sweet IR, Matschinsky FM. Mathematical model of β-cell glucose metabolism and insulin release. I. Glucokinase as glucosensor hypothesis. Am J Physiol. 1995;268:E775–88. 7733279 10.1152/ajpendo.1995.268.4.E775

[87] Detimary P, Dejonghe S, Ling Z, Pipeleers D, Schuit F, Henquin JC. The changes in adenine nucleotides measured in glucose-stimulated rodent islets occur in β cells but not in α cells and are also observed in human islets. J Biol Chem. 1998;273:33905–8. 9852040 10.1074/jbc.273.51.33905

[88] Schuit F, De Vos A, Farfari S, Moens K, Pipeleers D, Brun T, et al Metabolic fate of glucose in purified islet cells. Glucose-regulated anaplerosis in β cells. J Biol Chem. 1997;272:18572–9. 9228023 10.1074/jbc.272.30.18572

[89] Ravier MA, Rutter GA. Glucose or insulin, but not zinc ions, inhibit glucagon secretion from mouse pancreatic α-cells. Diabetes. 2005;54:1789–97. 15919801 10.2337/diabetes.54.6.1789

[90] Quesada I, Todorova MG, Soria B. Different metabolic responses in α-, β-, and δ-cells of the islet of Langerhans monitored by redox confocal microscopy. Biophys J. 2006;90:2641–50. 16399832 10.1529/biophysj.105.069906 PMC1403195

[91] Quoix N, Cheng-Xue R, Mattart L, Zeinoun Z, Guiot Y, Beauvois MC, et al Glucose and pharmacological modulators of ATP-sensitive K^+^ channels control [Ca^2+^]_c_ by different mechanisms in isolated mouse α-cells. Diabetes. 2009;58:412–21. 19008345 10.2337/db07-1298 PMC2628615

[92] MacDonald PE, De Marinis YZ, Ramracheya R, Salehi A, Ma X, Johnson PR, et al A K_ATP_ channel-dependent pathway within α cells regulates glucagon release from both rodent and human islets of Langerhans. PLoS Biol. 2007;5:e143. 17503968 10.1371/journal.pbio.0050143 PMC1868042

[93] Zhang Q, Ramracheya R, Lahmann C, Tarasov A, Bengtsson M, Braha O, et al Role of K_ATP_ channels in glucose-regulated glucagon secretion and impaired counterregulation in type 2 diabetes. Cell Metab. 2013;18:871–82. 24315372 10.1016/j.cmet.2013.10.014 PMC3851686

[94] Vieira E, Liu YJ, Gylfe E. Involvement of α_1_ and β-adrenoceptors in adrenaline stimulation of the glucagon-secreting mouse α-cell. Naunyn Schmiedebergs Arch Pharmacol. 2004;369:179–83. 14727006 10.1007/s00210-003-0858-5

[95] De Marinis YZ, Salehi A, Ward CE, Zhang Q, Abdulkader F, Bengtsson M, et al GLP-1 inhibits and adrenaline stimulates glucagon release by differential modulation of N- and L-type Ca^2+^ channel-dependent exocytosis. Cell Metab. 2010;11:543–53. 20519125 10.1016/j.cmet.2010.04.007 PMC4310935

[96] Ramracheya R, Ward C, Shigeto M, Walker JN, Amisten S, Zhang Q, et al Membrane potential-dependent inactivation of voltage-gated ion channels in α-cells inhibits glucagon secretion from human islets. Diabetes. 2010;59:2198–208. 20547976 10.2337/db09-1505 PMC2927942

[97] Wang JL, McDaniel ML. Secretagogue-induced oscillations of cytoplasmic Ca^2+^ in single β and α-cells obtained from pancreatic islets by fluorescence-activated cell sorting. Biochem Biophys Res Commun. 1990;166:813–18. 2405856 10.1016/0006-291x(90)90882-n

[98] Berts A, Ball A, Gylfe E, Hellman B. Suppression of Ca^2+^ oscillations in glucagon-producing α_2_-cells by insulin/glucagon and amino acids. Biochim Biophys Acta. 1996;1310:212–16. 8611635 10.1016/0167-4889(95)00173-5

[99] Göpel SO, Kanno T, Barg S, Weng XG, Gromada J, Rorsman P. Regulation of glucagon secretion in mouse α-cells by K_ATP_ channels and inactivation of TTX-sensitive Na^+^ channels. J Physiol. 2000;528:509–20. 11060128 10.1111/j.1469-7793.2000.00509.x PMC2270147

[100] Gromada J, Ma X, Hoy M, Bokvist K, Salehi A, Berggren PO, et al ATP-sensitive K^+^ channel-dependent regulation of glucagon release and electrical activity by glucose in wild-type and SUR1^-/-^ mouse α-cells. Diabetes. 2004;53:S181–9. 15561909 10.2337/diabetes.53.suppl_3.s181

[101] Linford B, Salehi A, Vergari E, Zhang Q, Rorsman P. Glucagon secretion from pancreatic α-cells. Ups J Med Sci. 2016;121:(In this issue). 10.3109/03009734.2016.1156789 PMC4900066 27044683

[102] Shiota C, Rocheleau JV, Shiota M, Piston DW, Magnuson MA. Impaired glucagon secretory responses in mice lacking the type 1 sulfonylurea receptor. Am J Physiol Endocrinol Metab. 2005;289:E570–7. 15941784 10.1152/ajpendo.00102.2005

[103] Miki T, Liss B, Minami K, Shiuchi T, Saraya A, Kashima Y, et al ATP-sensitive K^+^ channels in the hypothalamus are essential for the maintenance of glucose homeostasis. Nat Neurosci. 2001;4:507–12. 11319559 10.1038/87455

[104] Muñoz A, Hu M, Hussain K, Bryan J, Aguilar-Bryan L, Rajan AS. Regulation of glucagon secretion at low glucose concentrations: evidence for adenosine triphosphate-sensitive potassium channel involvement. Endocrinology. 2005;146:5514–21. 16123162 10.1210/en.2005-0637

[105] Bokvist K, Olsen HL, Høy M, Gotfredsen CF, Holmes WF, Buschard K, et al Characterisation of sulphonylurea and ATP-regulated K^+^ channels in rat pancreatic A-cells. Pflügers Arch. 1999;438:428–36. 10519134 10.1007/s004249900076

[106] Quesada I, Nadal A, Soria B. Different effects of tolbutamide and diazoxide in α-, β-, and δ-cells within intact islets of Langerhans. Diabetes. 1999;48:2390–7. 10580428 10.2337/diabetes.48.12.2390

[107] Allister EM, Robson-Doucette CA, Prentice KJ, Hardy AB, Sultan S, Gaisano HY, et al UCP2 regulates the glucagon response to fasting and starvation. Diabetes. 2013;62:1623–33. 23434936 10.2337/db12-0981 PMC3636632

[108] Manning Fox JE, Gyulkhandanyan AV, Satin LS, Wheeler MB. Oscillatory membrane potential response to glucose in islet β-cells: a comparison of islet-cell electrical activity in mouse and rat. Endocrinology. 2006;147:4655–63. 16857746 10.1210/en.2006-0424

[109] Hjortoe GM, Hagel GM, Terry BR, Thastrup O, Arkhammar PO. Functional identification and monitoring of individual α and β cells in cultured mouse islets of Langerhans. Acta Diabetol. 2004;41:185–93. 15660202 10.1007/s00592-004-0164-9

[110] Lieberman EM, Pascarella J, Brunder D, Hargittai PT. Effect of extracellular potassium on ouabain-sensitive consumption of high-energy phosphate by crayfish giant axons: a study of the energy requirement for transport in the steady state. J Neurochem. 1990;55:155–64. 2355216 10.1111/j.1471-4159.1990.tb08833.x

[111] Quoix N, Cheng-Xue R, Guiot Y, Herrera PL, Henquin JC, Gilon P. The GluCre-ROSA26EYFP mouse: a new model for easy identification of living pancreatic α-cells. FEBS Lett. 2007;581:4235–40. 17706201 10.1016/j.febslet.2007.07.068

[112] Li J, Shuai HY, Gylfe E, Tengholm A. Oscillations of sub-membrane ATP in glucose-stimulated beta cells depend on negative feedback from Ca^2+^. Diabetologia. 2013;56:1577–86. 23536115 10.1007/s00125-013-2894-0 PMC3671113

[113] Chow RH, Lund PE, Löser S, Panten U, Gylfe E. Coincidence of early glucose-induced depolarization with lowering of cytoplasmic Ca^2+^ in mouse pancreatic β-cells. J Physiol. 1995;485:607–17. 7562604 10.1113/jphysiol.1995.sp020756 PMC1158031

[114] Gylfe E. Glucose-induced early changes in cytoplasmic calcium of pancreatic β-cells studied with time-sharing dual-wavelength fluorometry. J Biol Chem. 1988;263:5044–8. 3281934

[115] Westerlund J, Gylfe E, Bergsten P. Pulsatile insulin release from pancreatic islets with non-oscillatory elevation of cytoplasmic Ca^2+^. J Clin Invest. 1997;100:2547–51. 9366569 10.1172/JCI119797 PMC508455

[116] Johansson H, Gylfe E, Hellman B. Cyclic AMP raises cytoplasmic calcium in pancreatic α_2_-cells by mobilizing calcium incorporated in response to glucose. Cell Calcium. 1989;10:205–11. 2550136 10.1016/0143-4160(89)90003-1

[117] Liu YJ, Gylfe E. Store-operated Ca^2+^ entry in insulin-releasing pancreatic β-cells. Cell Calcium. 1997;22:277–86. 9481478 10.1016/s0143-4160(97)90066-x

[118] Dyachok O, Gylfe E. Store-operated influx of Ca^2+^ in the pancreatic β-cells exhibits graded dependence on the filling of the endoplasmic reticulum. J Cell Sci. 2001;114:2179–86. 11493653 10.1242/jcs.114.11.2179

[119] Gylfe E. Carbachol induces sustained glucose-dependent oscillations of cytoplasmic Ca^2+^ in hyperpolarized pancreatic β cells. Pflügers Arch. 1991;419:639–43. 1788058 10.1007/BF00370308

[120] Tengholm A, Hellman B, Gylfe E. Glucose regulation of free Ca^2+^ in the endoplasmatic reticulum of mouse pancreatic beta cells. J Biol Chem. 1999;274:36883–90. 10601240 10.1074/jbc.274.52.36883

[121] Ravier MA, Daro D, Roma LP, Jonas JC, Cheng-Xue R, Schuit FC, et al Mechanisms of control of the free Ca^2+^ concentration in the endoplasmic reticulum of mouse pancreatic β-cells: interplay with cell metabolism and [Ca^2+^]_c_ and role of SERCA2b and SERCA3. Diabetes. 2011;60:2533–45. 21885870 10.2337/db10-1543 PMC3178295

[122] Tian G, Tepikin AV, Tengholm A, Gylfe E. cAMP induces stromal interaction molecule 1 (STIM1) puncta but neither Orai1 protein clustering nor store-operated Ca^2+^ entry (SOCE) in islet cells. J Biol Chem. 2012;287:9862–72. 22298778 10.1074/jbc.M111.292854 PMC3323006

[123] Bode HP, Weber S, Fehmann HC, Göke B. A nutrient-regulated cytosolic calcium oscillator in endocrine pancreatic glucagon-secreting cells. Pflügers Arch. 1999;437:324–34. 9914388 10.1007/s004240050786

[124] Läuger P. Electrogenic ion pumps. Sunderland, MA: Sinauer Associates Inc; 1991.

[125] Davies SL, Brown PD, Best L. Glucose-induced swelling in rat pancreatic α-cells. Mol Cell Endocrinol. 2007;264:61–7. 17112656 10.1016/j.mce.2006.10.005

[126] Boom A, Lybaert P, Pollet JF, Jacobs P, Jijakli H, Golstein PE, et al Expression and localization of cystic fibrosis transmembrane conductance regulator in the rat endocrine pancreas. Endocrine. 2007;32:197–205. 18040894 10.1007/s12020-007-9026-x

[127] Best L, Brown PD, Sener A, Malaisse WJ. Electrical activity in pancreatic islet cells: the VRAC hypothesis. Islets. 2010;2:59–64. 21099297 10.4161/isl.2.2.11171

[128] Sun G, da Silva Xavier G, Gorman T, Priest C, Solomou A, Hodson DJ, et al LKB1 and AMPKα1 are required in pancreatic alpha cells for the normal regulation of glucagon secretion and responses to hypoglycemia. Molecular Metabolism. 2015;4:277–86. 25830091 10.1016/j.molmet.2015.01.006 PMC4354920

[129] Watts M, Sherman A. Modeling the pancreatic α-cell: dual mechanisms of glucose suppression of glucagon secretion. Biophys J. 2014;106:741–51. 24507615 10.1016/j.bpj.2013.11.4504 PMC3944880

